# Aromaticity Determines the Relative Stability of Kinked vs. Straight Topologies in Polycyclic Aromatic Hydrocarbons

**DOI:** 10.3389/fchem.2018.00561

**Published:** 2018-11-20

**Authors:** Jordi Poater, Miquel Duran, Miquel Solà

**Affiliations:** ^1^ICREA, Barcelona, Spain; ^2^Departament de Química Inorgànica i Orgànica & IQTCUB, Universitat de Barcelona, Barcelona, Spain; ^3^Institut de Química Computacional i Catàlisi (IQCC) and Departament de Química, Universitat de Girona, Girona, Spain

**Keywords:** acenes, phenacenes, aromaticity, density functional theory (DFT), energy decomposition analysis (EDA), turn-upside-down approach

## Abstract

It is well-known that kinked phenacenes are more stable than their isomeric linear acenes, the archetypal example being phenanthrene that is more stable than anthracene by about 4–8 kcal/mol. In previous studies, the origin of the higher stability of kinked polycyclic aromatic hydrocarbons (PAHs) was found to be better π-bonding interactions, i.e., larger aromaticity, in kinked as compared to linear PAHs. Some years ago, however, Dominikowska and Palusiak (2011) found that dicationic linear anthracene is more stable than the dicationic kinked phenanthrene. Therefore, these authors showed that, in some cases, the linear topology in PAHs can be preferred over the kinked one. Our results using energy decomposition analyses in combination with the turn-upside-down approach show that the origin of the higher stability of dicationic anthracene is the same as in the neutral species, i.e., better π-bonding interactions. A similar result is found for the kinked and straight pyrano-chromenes. We conclude that the aromaticity is the driving force that determines the relative stability of kinked vs. straight topologies in PAHs.

## Introduction

The change in topology when going from linear anthracene to its isomeric kinked phenanthrene has important consequences. From an energetic point of view, phenanthrene is more stable than anthracene by about 4–8 kcal/mol (Balaban, 1980; Moyano and Paniagua, 1991, 1993; Behrens et al., 1994; Kato et al., 2002; Matta et al., 2003; Randić, 2003; Poater et al., 2007b). As compared to anthracene, phenanthrene has a higher ionization potential (by about 0.4 eV) (Boschi et al., 1974; Dabestani and Ivanov, 1999; Kato et al., 2002) and a reduced electron affinity (by ca. 0.5 eV) (Tschurl et al., 2006), as well as, a larger HOMO-LUMO gap as indicated by theoretical calculations (Kato et al., 2002; Poater et al., 2007b). Moreover, there is a blue shift in the S_1_ → S_0_ transition when going from anthracene to phenanthrene (Dabestani and Ivanov, 1999). All these observations indicate a higher kinetic and thermodynamic stability of phenanthrene with respect to anthracene. Still, both isomers undergo addition reactions usually at 9,10 position (Clar, 1964; Biermann and Schmidt, 1980; Wiberg, 1999). Theoretical calculations show that the ring currents are mainly localized in the central six-membered ring (6-MR) in anthracene, whereas for phenanthrene they are more intense in the outer 6-MRs (Anusooya et al., 1998; Ligabue et al., 1999; Steiner et al., 2002). These different ring currents lead to different NMR response and magnetizability values (Ligabue et al., 1999).

**Scheme 1:**
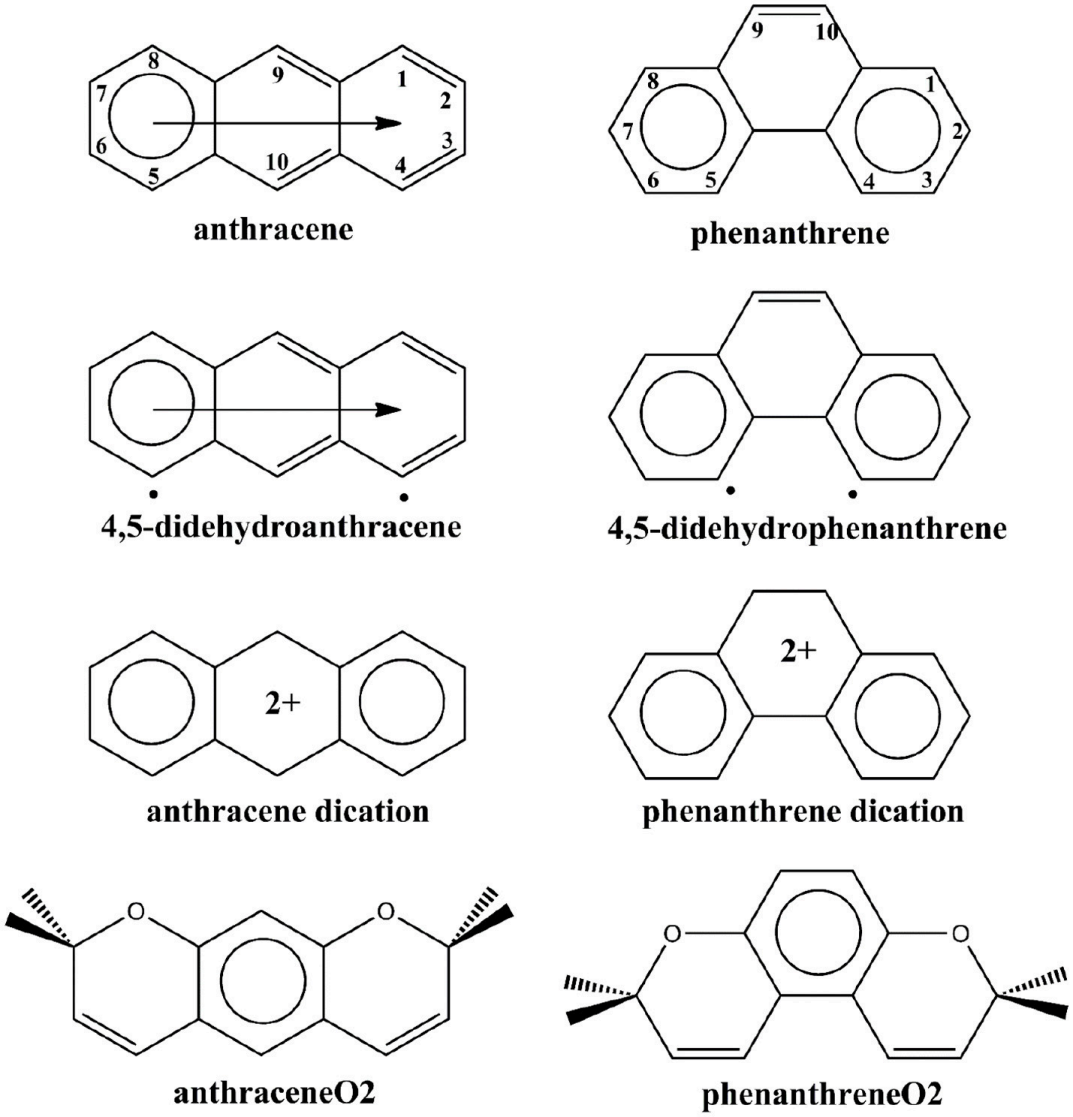
Clar's representation of neutral, 4,5-didehydro, and dicationic anthracene and phenanthrene, and straight and kinked tetramethylated-pyrano-chromenes. The neutral representation of anthracene and phenanthrene with the labeling of the different carbon atoms.

The origin of the larger relative stability of phenanthrene as compared to anthracene is controversial. Several authors (Matta et al., 2003; Bader, 2006; Wolstenholme and Cameron, 2006; Hernández-Trujillo and Matta, 2007; Eskandari and Van Alsenoy, 2014; Monteiro and Firme, 2014) based on the results from the quantum theory of atoms in molecules (QTAIM) consider that the H···H attraction between the hydrogen atoms of the bay region of phenanthrene (H4 and H5 attached to C4 and C5 in Scheme 1) justifies its higher stability. These two hydrogen atoms are about 5 kcal/mol stabilized with respect to hydrogen atoms in anthracene. This result was interpreted considering the presence of a H···H bonding interaction that contributes to the stabilization of phenanthrene by ca. 10 kcal/mol. Presence of this H···H stabilizing interaction would explain the larger stability of kinked phenacenes as compared to their isomeric linear acenes. It is worth mentioning that this interpretation differs from that of other authors that contemplate the existence of *London dispersion* stabilizations in hydrocarbons due to CH···HC weak interactions (Echeverría et al., 2011, 2017; Danovich et al., 2013).

**Scheme 2:**
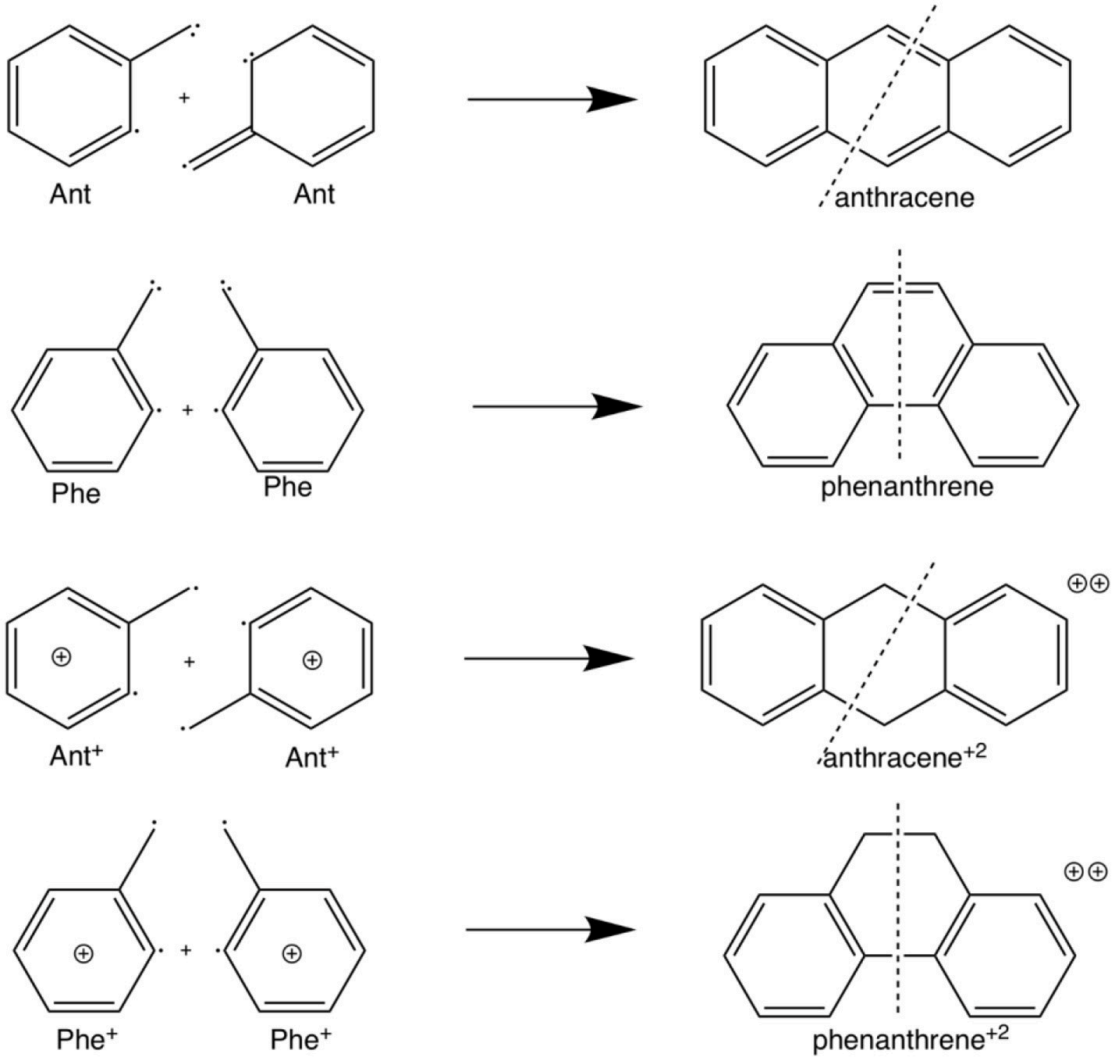
Fragmentation considered in the energy decomposition analysis performed in neutral and dicationic anthracene and phenanthrene.

Another more classical interpretation of the higher stability of phenanthrene derives from the analysis of the π-bonding interactions. Already in 1933, Pauling and Sherman found that the resonance of the π-system is more effective in phenanthrene than in anthracene (Pauling and Sherman, 1933). Later on, Dewar and De Llano (1969) as well as, Hess and Schaad (1971) confirmed these results by finding large resonance energies in phenanthrene as compared to anthracene (Cyranski, 2005). Fukui also explained the larger stability of phenanthrene by constructing the two benzenoids from naphthalene and butadiene fragments and showing that π-interactions are more stabilizing in phenanthrene (Fukui, 1982a,b). Clar's π-sextet model also indicates a larger stability of the π-system of phenanthrene by assigning two π-sextets to phenanthrene and only one migrating π-sextet to anthracene (see Scheme 1; Clar, 1972; Randić, 2003; Portella et al., 2005; Solà, 2013). More recently, some of us (Poater et al., 2007b), using energy decomposition analyses proved that π-electron interactions between two 2-methtriyl-phenyl fragments (**Ant** or **Phe** in Scheme 2) that form phenanthrene are more stabilizing than those generated in the formation of anthracene using the same fragments. Moreover, by comparing the energies of the triplet states 4,5-didehydrophenanthrene and 4,5-didehydroanthracene (Scheme 1), we also found that the 4,5-didehydrophenanthrene species is 5.2 kcal/mol more stable than 4,5-didehydroanthracene (Poater et al., 2007b), whereas at the same level of theory phenanthrene is only 4.2 kcal/mol more stable than anthracene. Moreover, comparison between different didehydrophenanthrenes in their triplet states indicate that 4,5-didehydrophenanthrene is particularly stable (Poater et al., 2007a). In our opinion, these results are a clear indication of the repulsive character of the H···H interaction between the bay H atoms in phenanthrene. Thus, our work confirmed the classical picture of better π-bonding causing phenanthrene's enhanced stability, despite the unfavorable H···H repulsion (Poater et al., 2006a,b) between the hydrogen atoms of the bay region. This interpretation was reinforced by means of natural bond orbital (NBO) (Alkorta et al., 2008; Weinhold et al., 2014) and quantum kinetic energy density analyses (Jacobsen, 2010). Remarkably, Grimme et al. (2009) provided conclusive experimental evidence supporting the traditional view of steric repulsion between the H atoms of the bay region of phenanthrene. Finally, in 2007, Martín Pendás et al. (2007) in an attempt to reconcile the orthodox QTAIM interpretation with the classical view, showed that bond paths in QTAIM are not necessarily a sign of attractive or repulsive interactions, but indications of the presence of preferred quantum-mechanical exchange channels.

In a more recent work, Dominikowska and Palusiak (2011), found that the anthracene dication is more stable than phenanthrene dication by about 16 kcal/mol. Therefore, by just removing two electrons from anthracene and phenanthrene, the relative stability of these two isomers is reversed. This result cannot be predicted in the framework of the conventional QTAIM H···H attractive interaction because this interaction should not be affected significantly by removing two π-electrons. On the other hand, it is easily understood by Clar's π-sextet model and the classical picture of H···H repulsion. Both anthracene and phenanthrene dications have two π-sextets (see Scheme 1). From the Clar model, the stabilization of the π-system is expected to be similar for the two C_14_H${}_{10}^{2+}$ isomers and, in this situation, the H···H steric repulsion should be the factor that makes phenanthrene dication less stable than anthracene dication. However, it is very important to take into account other factors not included in the Clar model to explain the phenanthrene dication reduction of stability. In particular, the loss of aromaticity due to partial delocalization of the positive charge to the external rings of phenanthrene plays also a very important role in explaining this reduction of stability (*vide infra*).

**Scheme 3:**
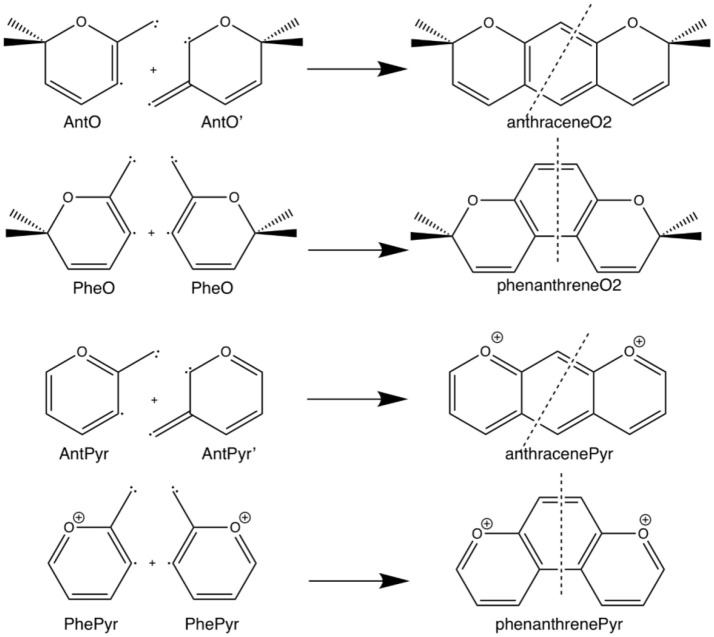
Fragmentation considered in the energy decomposition analysis performed in the pyrano-chromenes derivatives of anthracene and phenanthrene.

The example of the change in relative stabilities when going from neutral to dicationic phenanthrene and anthracene is an indication that the stability of the π-system, i.e., the aromaticity, plays a key role in determining the relative stability of kinked vs. straight topologies in polycyclic benzenoids. The main goal of this paper is to test this hypothesis by studying first the isomerization energies of the couples phenanthrene/anthracene and dicationic phenanthrene/anthracene, shown in Scheme 2. And next two couples of pyrano-chromenes: tetramethylated (linear 2,2,8,8-tetrametyl-2H,8H-pyrano[3,2-g]chromene–hereafter anthraceneO2–vs. kinked 3,3,8,8-tetramethyl-3,8-dihydropyrano[3,2-f]chromene–hereafter phenanthreneO2–) and dicationic pyrilium derived (linear pyrano[3,2-g]chromene-1,9-diium–hereafter anthracenePyr–vs. kinked pyrano[3,2-f]chromene-4,7-diium–hereafter phenanthrenePyr–), shown in Scheme 3. In the first pair of isomers, phenanthrene is more aromatic and more stable than anthracene, whereas in the second and third couples the aromaticity of the two isomers is expected to be similar because the number of π-sextets is the same for the two isomers and, therefore, it is likely that the straight isomer will be the most stable. For the last couple, we expect a behavior closer to that of anthracene/phenanthrene. Our investigation will be performed by means of an energy decomposition analysis of the Kohn-Sham density functional theory (DFT) wavefunction using the turn-upside-down approach (El-Hamdi et al., 2011, 2013) to analyze the studied isomerization energies. We anticipate here that our calculations show that the aromaticity of straight and kinked isomers is the main factor that determines the relative stability of kinked vs. straight topologies in PAHs.

## Materials and methods

All geometry optimizations were performed with the Gaussian 09 package (Frisch et al., 2009) by using the B3LYP hybrid density functional (Lee et al., 1988; Becke, 1993) and the 6-311++G(3df,3pd) basis set (Frisch et al., 1984) without symmetry constraints (Table S1). Analytical Hessians were computed to confirm that the optimized structures are indeed minima (zero imaginary frequencies).

Aromaticity was evaluated at the same level of theory by means of electronic-based aromaticity criteria (Poater et al., 2005; Feixas et al., 2015) such as the multicenter electron sharing indices (MCI) (Giambiagi et al., 2000; Bultinck et al., 2005), the *para* delocalization index (PDIs) (Poater et al., 2003), and the aromatic fluctuation index (FLU) (Matito et al., 2005), as well as, with the geometry-based harmonic oscillator model of aromaticity (HOMA) (Kruszewski and Krygowski, 1972; Krygowski, 1993) and the magnetic-based nucleus-independent chemical shift (NICS) obtained using the GIAO approximation (Cheeseman et al., 1996; Chen et al., 2005). MCIs provide a measure of electron sharing among the atoms considered, whereas delocalization indices (DIs) are two-center electron sharing indices (i.e., two-center MCIs) that measure electron sharing between two atoms (Fradera et al., 1999, 2002) and are required to calculate both PDI and FLU. Although several partitions can be used to define the atomic regions needed to calculate DIs and MCIs (Matito et al., 2006), we made use of the molecular partition based on the QTAIM (Bader, 1990, 1991). MCI, PDI, and FLU indices were obtained with the ESI-3D program (Matito et al., 2007; Matito, 2014). For MCI, PDI, and HOMA, the larger the values of a given ring, the higher its aromaticity; whereas for FLU, the closer to zero, the more aromatic; and for NICS, the more negative the NICS, the more aromatic the ring. Finally, we have also performed calculations of the anisotropy of the induced current density (ACID) (Herges and Geuenich, 2001; Geuenich et al., 2005) to analyze the ring-currents at the B3LYP/6-311+G(3df,3pd) level.

The bonding analysis was performed by means of an energy decomposition analysis (EDA), as implemented in ADF (te Velde et al., 2001). Calculations were done at the BLYP/TZ2P level, with the optimized geometries obtained at B3LYP/6-311++G(3df,3pd) level. The bonding analysis was carried out considering two quartet triradicals, fragment 1 (α*αα*) + fragment 2 (β*ββ*) (see Schemes 2, 3) for the formation of either anthracene or phenanthrene, as well as, of the tetramethylated and dicationic pyrilium pyrano-chromene isomers, whereas for the dicationic anthracene and phenanthrene two triplet diradicals, fragment 1 (αα) + fragment 2 (ββ) were employed. From the defined fragments, the bonding energy was decomposed into two major components (Equation 1; Bickelhaupt and Baerends, 2000; Fernández and Bickelhaupt, 2014; Bickelhaupt and Houk, 2017):

(1) $$\begin{array}{r} \Delta E=\Delta E_{\text{strain}}+\Delta E_{\text{int}} \end{array}$$

In this formula, the strain energy Δ*E*_strain_ is the amount of energy required to deform the separated triradical or diradical fragments from their equilibrium structure to the geometry that they acquire in the molecule. The interaction energy Δ*E*_int_ corresponds to the actual energy change when the prepared fragments are combined to form the overall molecule. It is analyzed in the framework of the Kohn-Sham MO model using a Morokuma-type (Kitaura and Morokuma, 1976; Morokuma, 1977) decomposition of the bonding energy into electrostatic interaction, exchange (or Pauli) repulsion, orbital interactions, and dispersion forces (Equation 2; Ziegler and Rauk, 1977):

(2) $$\begin{array}{r} \Delta E_{\text{int}}=\Delta V_{\text{elstat}}+\Delta E_{\text{Pauli}}+\Delta E_{\text{oi}}+\Delta E_{\text{disp}} \end{array}$$

The term Δ*V*_elstat_ corresponds to the classical electrostatic interaction between the unperturbed charge distributions of the prepared (i.e., deformed) fragments and is usually attractive. The Pauli repulsion, Δ*E*_Pauli_, comprises the destabilizing interactions between occupied MOs and it is responsible for the steric repulsion. The orbital interaction, Δ*E*_oi_, accounts for bond pair formation (interactions between singly occupied orbitals on one moiety with singly occupied orbitals on the other), charge transfer (donor–acceptor interactions between occupied orbitals of one fragment with unoccupied orbitals of the other, including the HOMO-LUMO interactions), and polarization (empty–occupied orbital mixing on one fragment due to the presence of another fragment). The orbital interaction energy can be decomposed into the contributions (σ and π contributions in our systems, i.e., Δ*E*_oi_ = Δ*E*${{}_{\text{oi}}}^{\sigma }$ + Δ*E*${{}_{\text{oi}}}^{\pi }$) from each irreducible representation Γ of the interacting system using the extended transition state (ETS) scheme developed by Ziegler and Rauk (1977). Finally, the ΔE_disp_ term takes into account the interactions that are due to dispersion forces.

In the EDA, open-shell fragments were treated with the spin-unrestricted formalism but, for technical reasons, spin-polarization was not included. This error causes the studied bond to become in the order of a few kcal·mol^−1^ too strong. To facilitate a straightforward comparison, the EDA results were scaled to match exactly the regular bond energies (the correction factor applied to all components of the EDA is consistently in the range 0.95–0.99 in all model systems and does therefore not affect trends).

## Results and discussion

This section is organized as follows. First, we discuss the geometry and isomerization energy of the eight systems analyzed. Second, we quantify the local aromaticity of their rings. Third, we perform energy decomposition analyses using the fragments as depicted in Schemes 2, 3. Finally, we discuss the H···H interaction in the bay region of neutral and cationic phenanthrene and phenanthreneO2 in the framework of the QTAIM theory.

### Geometry and isomerization energy

Phenanthrene is found to be 5.1 kcal mol^−1^ more stable than anthracene at the B3LYP/6-311++G(3df,3pd) level and 4.7 kcal mol^−1^ with the BLYP/TZ2P//B3LYP/6-311++G(3df,3pd) method. In our previous work, we found an energy difference of 4.2 kcal mol^−1^ using the BLYP/TZ2P methodology (Poater et al., 2007b). Interestingly, when two electrons are removed from each system, i.e., dicationic benzenoids are formed, then dicationic anthracene is 16.4 kcal mol^−1^ more stable than its dicationic phenanthrene counterpart. First and second adiabatic ionization potentials for anthracene are lower than for phenanthrene (anthracene: ^1st^IP = 163.6, ^2nd^IP = 270.6; phenanthrene: ^1st^IP = 175.2, ^2nd^IP = 280.6 kcal mol^−1^). For tetramethylated-pyrano-chromenes, anthraceneO2 is 4.2 kcal mol^−1^ more stable than its kinked isomer; whereas the opposite is found for the dicationic pyrilium derivatives, with kinked phenanthrenePyr more stable than anthracenePyr but by only 0.8 kcal mol^−1^.

**Figure 1:**
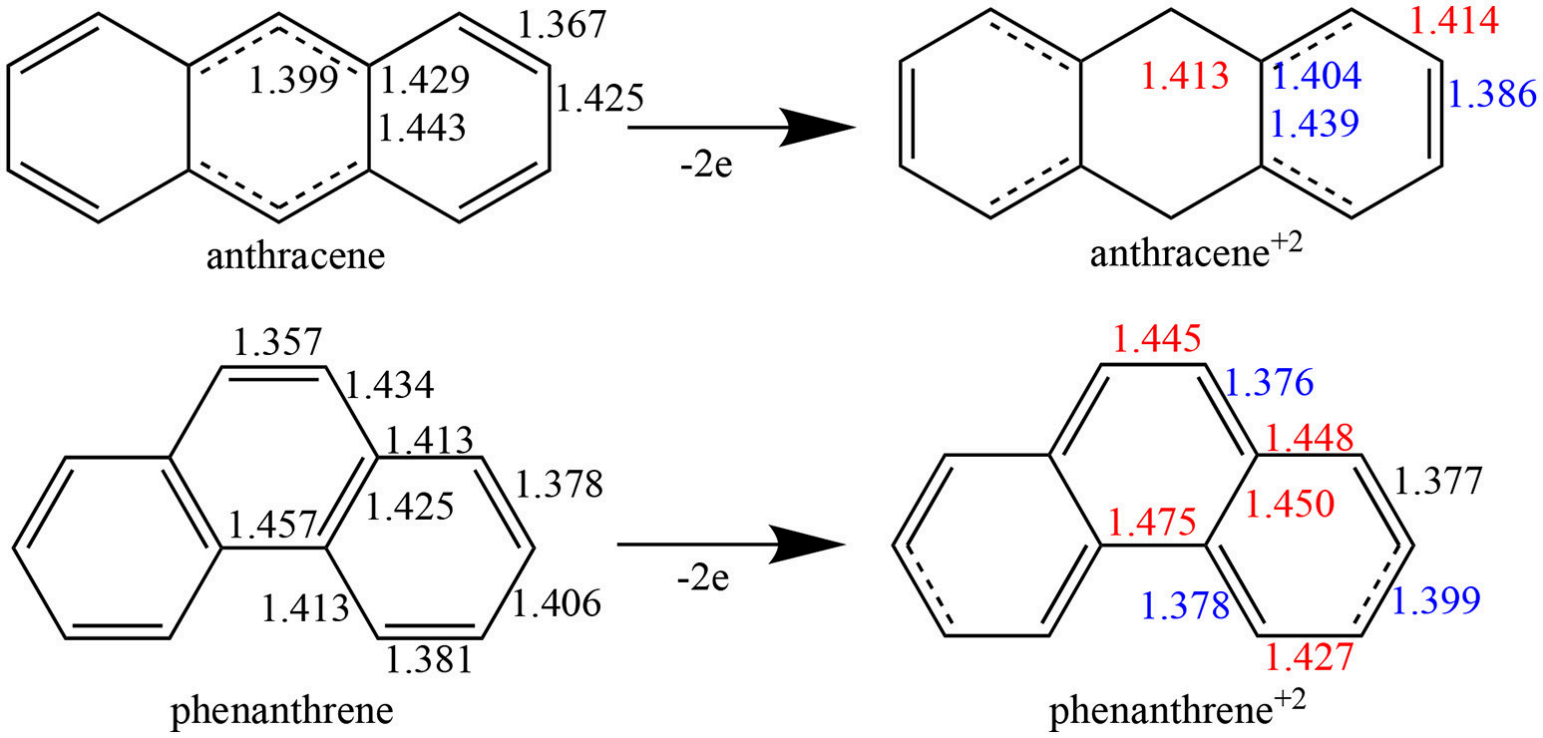
Bond lengths (in Å) of neutral and dicationic anthracene and phenanthrene. For the cationic systems, elongated bond lengths in red, and shortened in blue, compared to neutral ones.

Whereas, single and double bonds are mostly alternated in the three rings of both neutral anthracene and phenanthrene, when two electrons are removed (Figure 1) there is a reorganization, with some bonds elongated and others shortened in each isomer, with a localization of the π system mostly in the terminal rings. In particular, for dicationic anthracene, the π bonds become more localized in the two terminal rings in an alternated but delocalized way, whereas the C–C bonds of the central ring get more single-bond character, at variance with the neutral isomer (see schematic representation in Figure 1). On the other hand, for dicationic phenanthrene, the double bonds are localized in both terminal and central rings, but now the alternation (i.e., delocalization) is only observed for terminal ones, like for charged anthracene.

**Figure 2:**
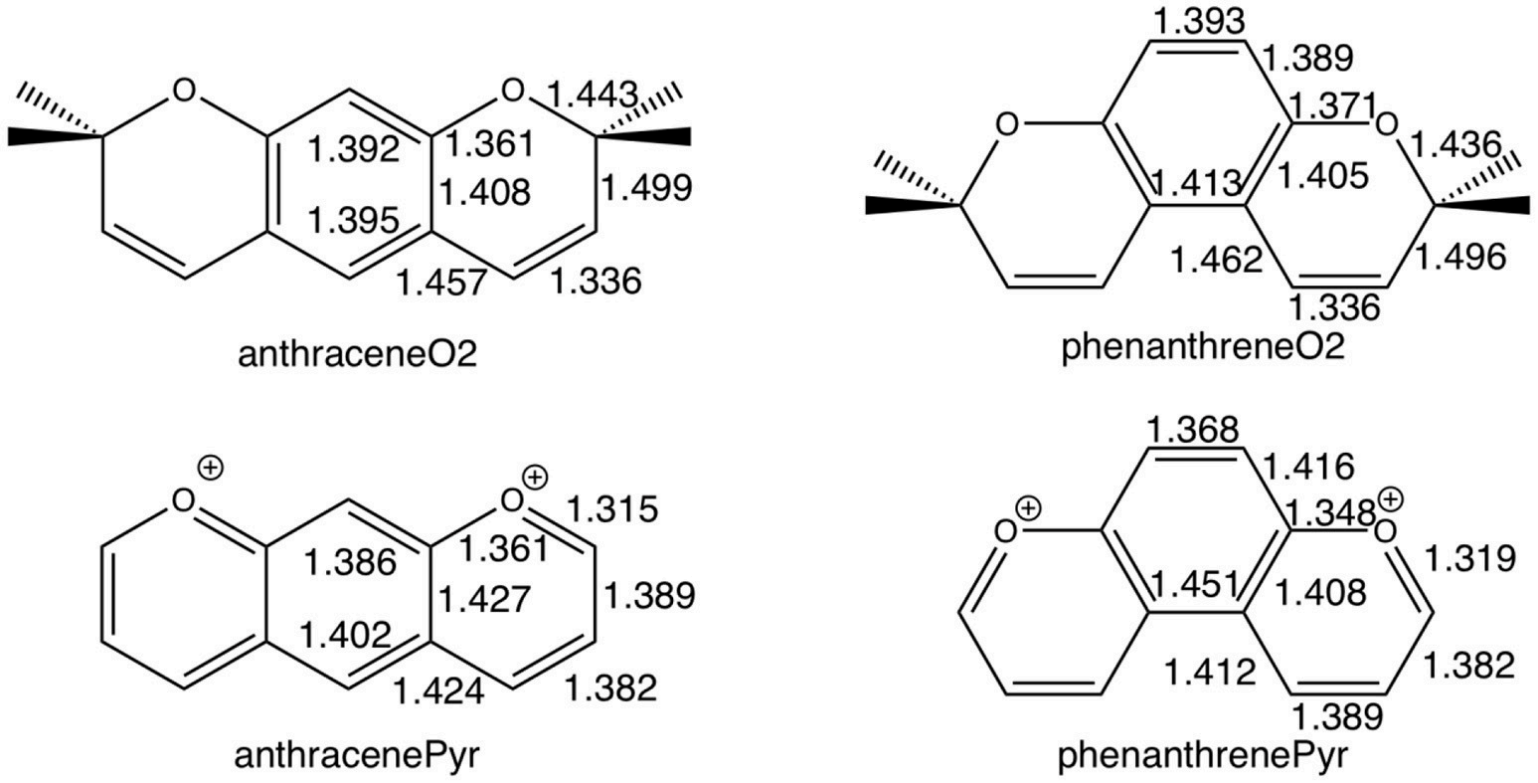
Bond lengths (in Å) of anthraceneO2 and phenanthreneO2 **(Top)** and anthracenePyr and phenanthrenePyr **(Bottom)**.

For tetramethylated-pyrano-chromenes, the methylation in the terminal rings causes a breaking of conjugation (Figure 2), with a unique double bond localized in each terminal ring. Meanwhile, the central ring in both isomers does present both single and double bonds. On the other hand, pyrilium derivatives again present conjugation along the three rings in both conformations (Figure 2).

### Aromaticity

The above discussion on the change in geometry and IPs when two electrons are removed from either anthracene or phenanthrene is directly connected to the aromaticity of these systems. Several aromaticity indices have been calculated, i.e., geometrical HOMA, electronic FLU, PDI, and MCI, and magnetic NICS. We will focus our discussion on the MCI results, as it has been previously shown in several tests undertaken that presents the best behavior among different indicators of aromaticity (Feixas et al., 2008, 2010). Table 1 encloses the values of all the aromaticity criteria. In the case of NICS, we list only NICS(0) in Table 1 because NICS(0)_zz_, NICS(1), and NICS(1)_zz_ given in Table S2 provide the same trends. First, in case of neutral isomers, the terminal rings of phenanthrene (MCI = 0.046) are clearly more aromatic than the central one (MCI = 0.018). The five aromaticity criteria point out in the same direction, thus supporting the two Clar π-sextets localized in the two terminal rings (Scheme 1). On the other hand, the aromaticity criteria show a similar aromaticity for terminal and central rings in anthracene (MCI = 0.029 and 0.027, respectively). In particular, HOMA, FLU, and NICS assign a somehow larger aromaticity to the central ring, whereas the inner and outer rings have an almost equivalent aromatic character according to both electronic-based criteria (PDI and MCI). This result agrees with its migrating Clar's π-sextet (Scheme 1). By comparison, terminal rings are more aromatic in phenanthrene, whereas the central one is more aromatic in anthracene (Szczepanik et al., 2018).

**Table 1:** Geometric HOMA, electronic FLU, PDI (in e^−^), and MCI (in e^−^), and magnetic NICS (in ppm) aromaticity criteria for anthracene and phenanthrene and derived species.

	**Central ring**					**Terminal ring**				
	**HOMA**	**FLU**	**PDI**	**MCI**	**NICS**	**HOMA**	**FLU**	**PDI**	**MCI**	**NICS**
Anthracene	0.719	0.010	0.065	0.027	−11.4	0.629	0.016	0.065	0.029	−7.3
Phenanthrene	0.461	0.020	0.046	0.018	−5.6	0.868	0.005	0.081	0.046	−8.5
Anthracene^2+^	0.672	0.017	0.026	0.013	16.5	0.813	0.009	0.050	0.028	11.1
Phenanthrene^2+^	0.197	0.026	0.033	0.010	23.8	0.601	0.016	0.046	0.019	26.8
AnthraceneO2	0.961	0.007	0.071	0.039	−6.7	−0.937	na	0.022	0.002	4.2
PhenanthreneO2	0.946	0.006	0.076	0.042	1.2	−0.924	na	0.023	0.002	2.8
AnthracenePyr	0.854	0.013	0.065	0.028	−12.5	0.582	na	0.053	0.015	−5.0
PhenanthrenePyr	0.713	0.019	0.049	0.022	−8.2	0.710	na	0.063	0.020	−3.7

Considering the Platt's perimeter model (Platt, 1949), neutral isomers follow 4n+2 Hückel's rule, with 14 π electrons, whereas the charged ones does not, because they have 12 π electrons, thus they should be considered antiaromatic based on this model. Then, once two electrons are removed, dicationic phenanthrene shows a clear reduction of aromaticity in both types of rings (MCI = 0.019 and 0.010 for terminal and central rings, respectively). Despite their reduction, terminal rings continue being somewhat aromatic, as proven by all criteria, except of NICS. The decrease of aromaticity also happens for the central ring, which might be considered non-aromatic. On the other hand, dicationic anthracene presents a different behavior: the terminal (MCI = 0.028) rings are now equally (PDI and MCI) or slightly more aromatic (HOMA and FLU) than in the neutral species, whereas the aromaticity of the central ring (MCI = 0.013) is slightly reduced. Both terminal and central rings of dicationic anthracene are more aromatic than for dicationic phenanthrene. Further, in both cases, the terminal rings are more aromatic than the central one, also in agreement with what is expected based on Clar's rule (Scheme 1). ACID plots (Figure S1) support the aforementioned changes in aromaticity when going from neutral to cationic phenanthrene and anthracene. Anthracene shows diamagnetic ring-currents in the inner and outer rings that become somewhat more intense in the outer rings when two electrons are removed. On the other hand, the ACID plot of phenanthrene indicates more aromatic outer than inner rings that lose aromaticity in the dicationic form. However, it must be pointed out the dependence of ACID on the location of the integration plane (Fliegl et al., 2016).

**Figure 3:**
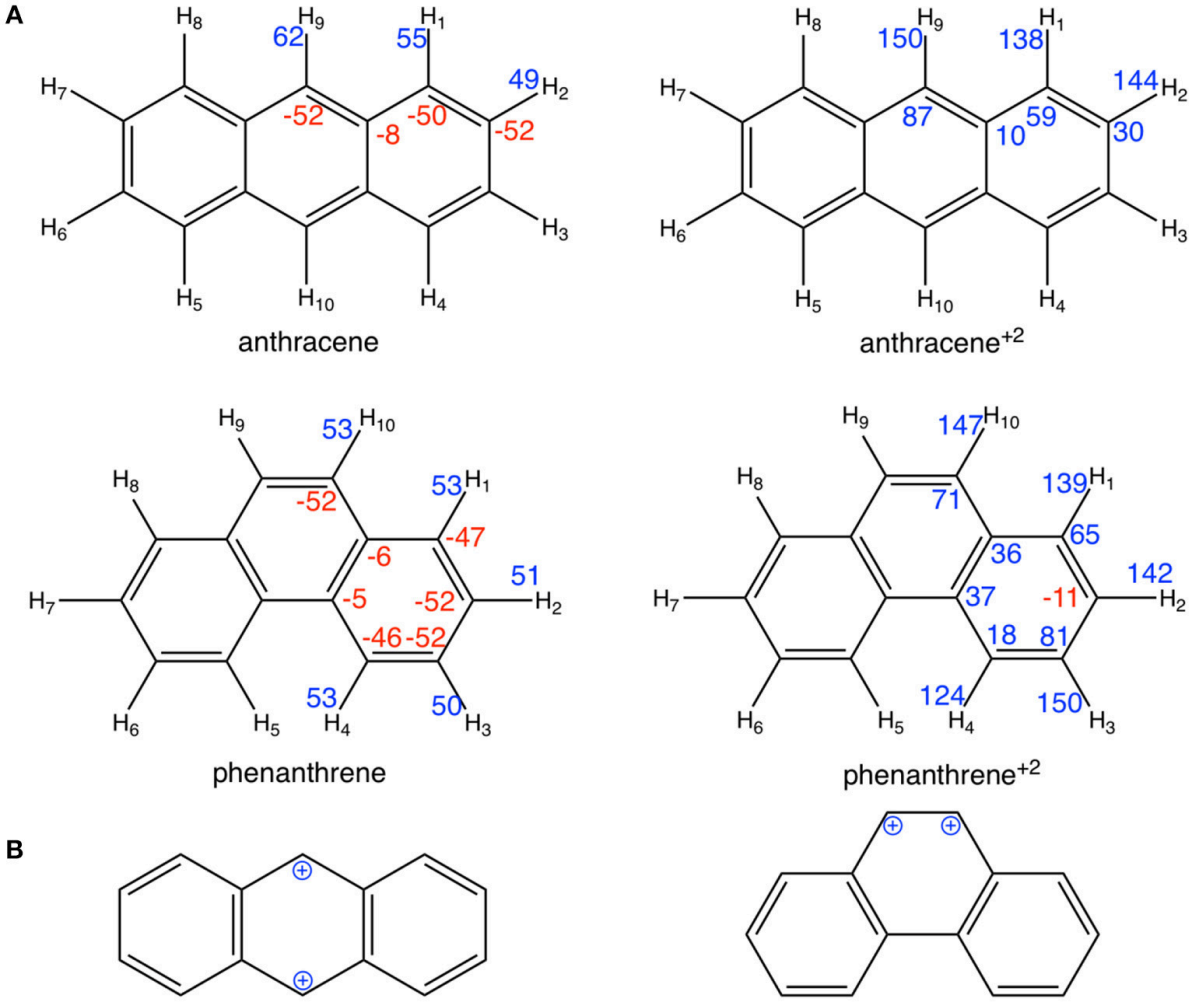
**(A)** VDD charges (in me^−^) for neutral and cationic anthracene and phenanthrene; **(B)** schematic representation of the benzyl cations for cationic anthracene and phenanthrene. Negative charges in red and positive in blue.

However, at variance with the prediction of the Clar model of Scheme 1, Voronoi deformation density (VDD) charges in Figure 3 show that, when the two electrons are removed from both anthracene and phenanthrene, the atoms of the terminal rings experience the largest loss of electronic charge (more positively charged atoms). More importantly, the Clar valence structure of anthracene dication corresponds to two benzyl cations located in *para* position, whereas that for phenanthrene dication corresponds to a vicinal dication (Figure 3B). These expected models are supported by the VDD charges (Figure 3A) with +87 me^−^ for carbons in *para* in anthracene^+2^ and +71 me^−^ for carbons in *ortho* in phenanthrene^+2^. The positive charges in *ortho* cause an important electrostatic repulsion that is reduced by partial delocalization of the positive charge to outer rings, thus decreasing the aromaticity of these rings. In this way, we can justify the loss of aromaticity of the outer rings of phenanthrene when two electrons are removed.

From the aromaticity analysis, in case of neutral systems the higher stability of the kinked isomer correlates with its larger aromaticity. On the other hand, for charged systems, despite both isomers present a closer aromatic character; anthracene dication is more aromatic, which also correlates with its larger stability. These results are in agreement with previous work by Dominikowska and Palusiak (2011). Furthermore, these trends in aromaticity also explain the ionization potentials discussed in the previous section, being smaller for anthracene as the loss of two electrons in this case transforms the less aromatic neutral system into the most aromatic cationic benzenoid.

With respect to the third set of isomers, i.e., tetramethylated-pyrano-chromene derivatives, the dimethylation of each terminal ring causes these rings to be non-aromatic (MCI = 0.002 for both isomers), whereas the central ring is clearly aromatic (MCI = 0.039 and 0.042 for anthraceneO2 and phenanthreneO2, respectively). All aromaticity criteria point out a similar aromaticity for both isomers, which does not explain the larger stability of the linear one. So, further analysis is required in order to understand the change in the relative stability of the linear and kinked isomers when we move from neutral to dicationic species. Finally, the behavior is opposite for the pyrilium derived systems, as in this case phenanthrenePyr is slightly more stable than anthracenePyr, which is likely to be because the differences in aromaticity are lower for these systems than for the couple anthracene/phenanthrene. In particular, the outer rings in phenanthrenePyr with MCI = 0.020 are significantly less aromatic than their analog rings in phenanthrene (MCI = 0.046).

### Energy decomposition analysis

**Figure 4:**
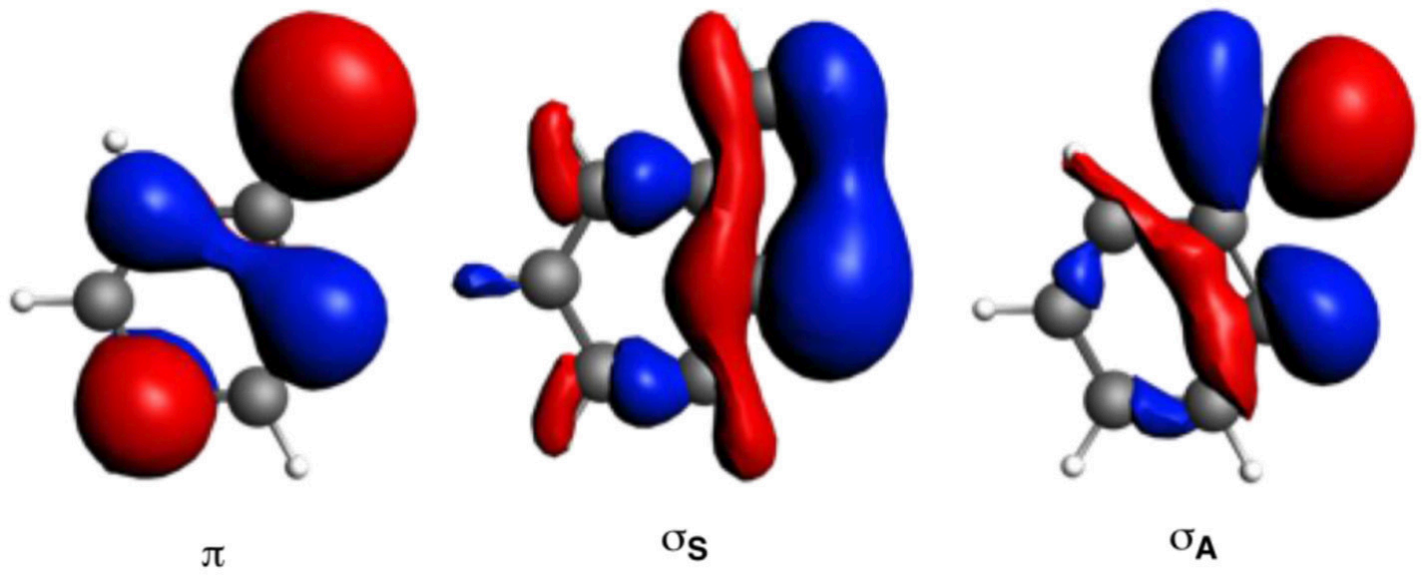
Singly-occupied molecular orbitals (SOMOs) of one fragment to build anthracene by means of the EDA analysis.

The larger stability of phenanthrene than anthracene, and the opposite for their corresponding dicationic systems, can be understood by means of a quantitative Kohn-Sham MO analysis complemented with an energy decomposition analysis (EDA, Table 2). We have to mention that the EDA of the anthracene and phenanthrene was already discussed in detail in our previous work (Poater et al., 2007b). Here, we briefly summarize the main results and provide the values obtained at the BLYP/TZ2P//B3LYP/6-311++G(3df,3pd) level of theory. Both phenanthrene and anthracene can be built from two identical 2-methtriyl-phenyl fragments (Scheme 2) in their quadruplet state. The three unpaired electrons are located in three singly occupied molecular orbitals (Figure 4). These are the σ_A_ and σ_S_ orbitals in the σ electron system and the π orbital in the π electron system. The two former are antisymmetric and symmetric regarding the sign of their large-amplitude lobes. The more favorable total bonding energy for phenanthrene (ΔΔE = −4.7 kcal mol^−1^) is due to the interaction energy (ΔΔE_int_ = −4.8 kcal mol^−1^), as the deformation of the fragments is almost the same in both cases (ΔΔE_strain_ = 0.1 kcal mol^−1^). ΔE_int_ can be further decomposed as in Equation (2). The more favorable ΔE_int_ is mainly attributed to Pauli repulsion (ΔΔE_Pauli_ = −16.1 kcal mol^−1^), whereas both attractive electrostatic and orbital interaction terms are more stable in anthracene (ΔΔV_elstat_ = 9.0 and ΔΔE_oi_ = 2.9 kcal mol^−1^). However, it has been previously proven that this observation is misleading, as it does not only reflect the pure changes in bonding because of “flipping” the two fragments to form either anthracene or phenanthrene, but it also contains the effect of structural relaxation that is induced by the changes in intrinsic bonding which also masks the latter (Bickelhaupt et al., 2002; Bickelhaupt and Baerends, 2003; Poater et al., 2007b). It has been shown that a system with larger Pauli repulsion may lead to an equilibrium structure with a longer bond in which ΔE_Pauli_ is lower than in the equilibrium structure of the sterically less demanding equivalent system. Thus, the differences between anthracene and phenanthrene cannot be only analyzed based on their equilibrium geometries, as we may get erroneous conclusions. To overcome this issue, the bonding analysis in a deformed phenanthrene has been performed by taking the same fragments as in anthracene, but just flipped, i.e., turn-upside-down approach (El-Hamdi et al., 2011), with the two formed C–C bonds kept fixed to the equilibrium distance of anthracene (formed C–C bonds: 1.399 Å). This modified phenanthrene (phenanthrene${{}_{\text{Ant}}}^{\text{Ant}}$) is still more stable than anthracene (ΔΔE = −1.2 kcal mol^−1^), although less than phenanthrene in its equilibrium geometry. The only difference between anthracene${{}_{\text{Ant}}}^{\text{Ant}}$ and phenanthrene${{}_{\text{Ant}}}^{\text{Ant}}$ is the connection of the two fragments, i.e., the topology. Phenanthrene${{}_{\text{Ant}}}^{\text{Ant}}$ presents almost the same steric repulsion between the two fragments as anthracene${{}_{\text{Ant}}}^{\text{Ant}}$ (ΔΔE_Pauli_ = −0.6 kcal mol^−1^), so it is not the determinant term any longer. The difference is mainly due to the π orbital interaction (ΔΔE${{}_{\text{oi}}}^{\pi }$ = −1.5 kcal mol^−1^). As can be seen in Figure 4, the π SOMO of the 2-methtriyl-phenyl fragment has small coefficients in the ring and a large one on the exocyclic carbon. A better π-π overlap is achieved when the two exocyclic lobes overlap and this occurs in phenanthrene. On the other hand, ΔΔE${{}_{\text{oi}}}^{\text{σ}}$ = 0.9 kcal mol^−1^ and this result is important because if the stabilizing H···H bonding in the bay region of phenanthrene would exist, as previously stated based on QTAIM, then ΔE${{}_{\text{oi}}}^{\sigma }$ would favor phenanthrene topology.

**Table 2:** Energy decomposition analysis (EDA) data for anthracene and phenanthrene (in kcal mol^−1^), and the corresponding turn-upside-down derivatives.

	**Anthracene${{}_{\text{Ant}}}^{\text{Ant}}$**	**Phenanthrene${{}_{\text{Phe}}}^{\text{Phe}}$**		**Phenanthrene${{}_{\text{Ant}}}^{\text{Phe}}$**		**Phenanthrene${{}_{\text{Ant}}}^{\text{Ant}}$**
	**Ant**+**Ant**	**Phe**+**Phe**	*ΔΔ**E***	**Ant**+**Ant**	*ΔΔ**E***	**Ant**+**Ant**	ΔΔ**E**
ΔE_Pauli_	583.7	567.6	−16.1	574.9	−8.8	583.1	−0.6
ΔV_elstat_	−364.6	−355.7	9.0	−358.3	6.3	−363.6	1.0
ΔE${{}_{\text{oi}}}^{\sigma }$	−419.0	−413.6	5.5	−415.6	3.4	−418.3	0.7
ΔE${{}_{\text{oi}}}^{\text{π}}$	−86.7	−89.2	−2.5	−89.3	−2.6	−88.2	−1.5
ΔE_oi_	−505.7	−502.8	2.9	−504.9	0.8	−506.5	−0.8
ΔE_disp_	−9.2	−9.7	−0.5	−9.7	−0.5	−9.9	−0.7
ΔE_int_	−295.8	−300.6	−4.8	−298.1	−2.3	−296.9	−1.1
ΔE_strain_	15.8	15.9	0.1	15.8	0.0	15.8	0.0
ΔE	−280.0	−284.7	−4.7	−282.3	−2.3	−281.2	−1.2

The next step consisted of using the same two fragments, but now with the formed C–C equal to those of phenanthrene (formed C–C bonds: 1.357 and 1.457 Å). This new geometry (phenanthrene${}_{\text{Ant}}^{\text{Phe}}$) already gives a lower Pauli repulsion (ΔΔE_Pauli_ = −8.8 kcal mol^−1^), but more importantly, together with a larger stabilization of the π orbital interaction (ΔΔE${{}_{\text{oi}}}^{\text{π}}$ = −2.6 kcal mol^−1^). The final step involves going to the fully relaxed phenanthrene, which clearly shows an even increased reduction of ΔE_Pauli_, as stated above, due to the bending of the C–H bonds in the bay region. So, based on these model systems, the more efficient bonding in the π-electron system in phenanthrene is responsible of its larger stability compared to its linear isomer, which is supported by its larger aromaticity. Such larger ΔΔE${{}_{\text{oi}}}^{\pi }$ for phenanthrene is also supported by the better overlap between the π SOMO of each fragment (0.243 vs. 0.196 for relaxed phenanthrene and anthracene, respectively; in case of phenanthrene${{}_{\text{Ant}}}^{\text{Phe}}$ and phenanthrene${{}_{\text{Ant}}}^{\text{Ant}}$, the overlaps are 0.236 and 0.230, respectively).

Now, as stated above, dicationic anthracene becomes more aromatic than charged phenanthrene, especially due to the decrease of aromaticity of the latter. However, despite their closer aromatic character, dicationic anthracene is more stable than phenanthrene by 16.2 kcal mol^−1^ (ΔΔE), with a larger difference than between the neutral isomers (see Table 3). From the EDA analysis, the strain energy is smaller for phenanthrene dication, which gives a ΔΔE_int_ = 22.2 kcal mol^−1^ in favor of dicationic anthracene. Like in the previous case, Pauli repulsion is smaller in charged phenanthrene (ΔΔE_Pauli_ = −64.7 kcal mol^−1^), however now it cannot compensate the more stable electrostatic and orbital interaction terms (ΔΔV_elstat_ = 31.5 and ΔΔE_oi_ = 55.8 kcal mol^−1^) of charged anthracene. Electrostatic interactions for the charged species are smaller than for the neutral anthracene and phenanthrene, as expected from the fact that the two approaching fragments in charged systems bear a positive charge. Interestingly, in dicationic anthracene and phenanthrene both the ΔΔE${{}_{\text{oi}}}^{\text{σ}}$ and ΔΔE${{}_{\text{oi}}}^{\text{π}}$ favor the straight isomer. As done before, the turn-upside-down approach has also been applied for these species to better understand the larger stability of dicationic anthracene vs. its kinked isomer. A deformed phenanthrene from the same fragments as for anthracene is built, first with the same new C–C bonds as those of dicationic anthracene (formed C–C bonds: 1.413 Å, phenanthrene^2+^_Ant_^Ant^), and next with the new C–C bonds as those of relaxed dicationic phenanthrene (formed C–C bonds: 1.445 and 1.475 Å, phenanthrene^2+^_Ant_^Phe^). Again, phenanthrene^2+^_Ant_^Ant^ and anthracene^2+^_Ant_^Ant^ differ only in the topology. Phenanthrene^2+^_AntO_^AntO^ is less stable than relaxed anthracene by 24.5 kcal mol^−1^. Pauli repulsion slightly favors the anthracene dication (ΔΔE_Pauli_ = 4.5 kcal mol^−1^), but the main contribution comes from more stabilizing orbital interactions (ΔΔE_oi_ = 18.3 kcal mol^−1^) both σ- (ΔΔE${{}_{\text{oi}}}^{\text{σ}}$ = 0.9 kcal mol^−1^) and π-orbital interactions (ΔΔE${{}_{\text{oi}}}^{\text{π}}$ = 17.4 kcal mol^−1^). Because the SOMOs σ are more or less the same in the neutral and charged species (Figure S2), the ΔΔE${{}_{\text{oi}}}^{\text{σ}}$ stabilization is basically equal (difference of only 0.2 kcal mol^−1^). For the π-system, the situation is totally different (Figure S3). In the neutral systems, there is a SOMO π that interacts better in phenanthrene topology than in anthracene one favoring the kinked isomer. In charged species, the fragments contain 6π electrons in three occupied π-MOs. Now, the ΔE${{}_{\text{oi}}}^{\text{π}}$ contains neither bond pair formation nor charge transfer interaction, only polarization. We consider that polarization is more favorable in the linear topology because positive charges are located mainly in the outer rings and these rings are more separated in the anthracene dication (Figure S4). Once the new C–C distances are changed to those of relaxed charged phenanthrene (phenanthrene^2+^_Ant_^Phe^), the terms are already very close to those of fully relaxed dicationic phenanthrene. So, despite Pauli repulsion is reduced, electrostatic and orbital interaction terms are less attractive in phenanthrene^2+^_Ant_^Phe^ than phenanthrene^2+^_Ant_^Ant^.

**Table 3:** Energy decomposition analysis (EDA) data for dicationic anthracene and phenanthrene (in kcal mol^−1^), and the corresponding turn-upside-down derivatives.

	**Anthracene^2+^_Ant_^Ant^**	**Phenanthrene^2+^_Phe_^Phe^**		**Phenanthrene^2+^_Ant_^Phe^**		**Phenanthrene^2+^_Ant_^Ant^**
	**Ant**^+^+**Ant**^+^	**Phe**^+^+**Phe**^+^	*ΔΔ**E***	**Ant**^+^+**Ant**^+^	*ΔΔ**E***	**Ant**^+^+**Ant**^+^	*ΔΔ**E***
ΔE_Pauli_	539.5	474.8	−64.7	472.1	−67.4	544.0	4.5
ΔV_elstat_	−236.5	−205.0	31.5	−201.2	35.3	−234.1	2.4
ΔE${{}_{\text{oi}}}^{\sigma }$	−421.9	−395.6	26.3	−394.5	27.4	−421.0	0.9
ΔE${{}_{\text{oi}}}^{\pi }$	−84.8	−55.4	29.4	−57.7	27.1	−67.4	17.4
ΔE_oi_	−506.7	−451.0	55.8	−452.1	54.6	−488.4	18.3
ΔE_disp_	−9.2	−9.5	−0.3	−9.7	−0.5	−9.9	−0.7
ΔE_int_	−212.9	−190.7	22.2	−190.9	22.0	−188.4	24.5
ΔE_strain_	19.5	13.5	−6.0	19.5	0.0	19.5	0.0
ΔE	−193.4	−177.2	16.2	−171.4	22.0	−168.9	24.5

Overall, our results prove that also for dicationic isomers, the ΔE${{}_{\text{oi}}}^{\text{π}}$ term plays a critical role in determining the relative isomeric energies. This term favors the kinked structure for neutral and the linear for dicationic anthracene and phenanthrene.

Next, we analyze the pair of kinked and straight tetramethylated-pyrano-chromene isomers having both only one single Clar sextet. These isomers can also be formed from the same fragments (Scheme 3). AnthraceneO2 presents a lower total binding energy than for phenanthrene isomer by 6.2 kcal mol^−1^ (ΔΔE), from which 3.5 kcal mol^−1^ comes from the strain energy and 2.7 kcal mol^−1^ from the interaction energy. Further, again the phenanthrene derivative has a lower Pauli repulsion (ΔΔE_Pauli_ = −33.8 kcal mol^−1^), which is compensated by more attractive electrostatic and orbital interactions in anthraceneO2 (ΔΔV_elstat_ = 18.9 and ΔΔE_oi_ = 18.6 kcal mol^−1^). In this case, the more stable ΔE_oi_ for anthraceneO2 mainly comes from the σ instead of the π interactions between the MOs of the two fragments, but like in the dications both favor the linear isomer. The ΔΔE${{}_{\text{oi}}}^{\text{π}}$ = 4.2 kcal mol^−1^ favoring the anthraceneO2 isomer, proves its determinant role at assigning the larger stability to either the kinked acene (neutral) or the linear one (dicationic and tetramethylated-pyrano-chromene derivatives). As for the neutral systems, this more favorable ΔΔE${{}_{\text{oi}}}^{\text{π}}$ for anthraceneO2 is supported by the overlaps between the π SOMOs of each fragment (0.228 vs. 0.247 for relaxed phenanthreneO2 and anthraceneO2, respectively). For phenanthreneO2 and anthraceneO2, the overlaps between the π SOMOs do not differ much in the two topologies because in the AntO fragment the contribution of the π SOMO in the ring and in the exocyclic carbon are very similar (see Figure S5).

Likewise, we applied the turn-upside-down approach to tetramethylated-pyrano-chromene derivatives. Unfortunately, it cannot be applied straight because the presence of the oxygen in the terminal rings causes that the fragment does not have the C2 axis to get the correct fragment when turned-upside-down in order to build the other isomer. So, instead of breaking anthraceneO2 system in two fragments (AntO + AntO') and rotating one of them, we need twice the same fragment (AntO), but one rotated, to build phenanthreneO2${{}_{\text{AntO}}}^{\text{AntO}}$ isomer (formed C–C bonds: 1.392 Å) and phenanthreneO2${{}_{\text{AntO}}}^{\text{PheO}}$ (formed C–C bonds: 1.393 and 1.413 Å) isomer, as above (Table 4). PhenanthreneO2${{}_{\text{AntO}}}^{\text{AntO}}$ is 7.0 kcal mol^−1^ less stable than anthraceneO2, which again is due to less stabilizing electrostatic and orbital interactions, which cannot compensate its lower Pauli repulsion. Then, in case of phenanthreneO2${{}_{\text{AntO}}}^{\text{PheO}}$, the values are already very close to phenanthreneO2, and the behavior very similar to that of dicationic systems, i.e., despite Pauli repulsion is reduced, electrostatic and orbital interaction terms are less attractive. Just for comparison, the overlaps between the π SOMOs in case of phenanthreneO2^2+^_AntO_^PheO^ and phenanthreneO2^2+^_AntO^AntO^_ are 0.226 and 0.230, respectively, to be compared to the discussed above of 0.247 for anthraceneO2.

**Table 4:** Energy decomposition analysis (EDA) data for tetramethylated-pyrano-chromenes (in kcal mol^−1^), and the corresponding turn-upside-down derivative.

	**AnthraceneO2_AntO^AntO^_**	**PhenanthreneO2_PheO^PheO^_**		**PhenanthreneO2_AntO^PheO^_**		**PhenanthreneO2_AntO^AntO^_**
	**AntO**+**AntO'**	**PheO**+**PheO**	*ΔΔ**E***	**AntO**+**AntO**	*ΔΔ**E***	**AntO**+**AntO**	*ΔΔ**E***
ΔE_Pauli_	576.3	542.5	−33.8	544.7	−31.7	566.4	−9.9
ΔV_elstat_	−360.4	−341.6	18.9	−342.6	17.9	−353.0	7.4
ΔE_oi^σ^_	−422.6	−408.1	14.4	−407.4	15.2	−415.1	7.5
ΔE_oi^π^_	−92.1	−87.9	4.2	−87.2	4.9	−90.3	1.8
ΔE_oi_	−514.6	−496.1	18.6	−494.6	20.0	−505.4	9.2
ΔE_disp_	−8.4	−9.3	−0.9	−9.4	−1.0	−9.5	−1.1
ΔE_int_	−307.1	−304.4	2.7	−302.0	5.2	−301.5	5.7
ΔE_strain_	15.5	19.0	3.5	16.8	1.3	16.8	1.3
ΔE	−291.6	−285.4	6.2	−285.2	6.5	−284.7	7.0

For the last couple of systems, i.e., anthracenePyr vs. phenanthrenePyr (Table 5 and Scheme 3), the latter appears to be only 0.9 kcal mol^−1^ than the former (ΔΔE). This difference arises due to more favorable ΔE_int_ term of the kinded system (ΔΔE_int_ = −3.2 kcal mol^−1^), despite its unfavorable strain energy (ΔΔE_strain_ = 2.2 kcal mol^−1^). At difference with the previous couple of pyrano-chromene systems, now the more favorable ΔV_elstat_ and ΔE_oi_ of the linear system cannot compensate its much larger ΔE_Pauli_, and, for this reason, phenanthrenePyr is more stable. From the turn-upside-down approach we can observe that when phenanthrenePyr is built from the same fragments of anthracenePyr, i.e., phenanthrenePyr _AntPyr_^AntPyr^ or phenanthreneO2_AntPyr_^PhePyr^, Pauli repulsion increases in phenanthrenePyr and causes the kinked system to be less stable, proving the determinant role of ΔE_Pauli_. At variance with what is observed in phenanthrene, the ΔE_oi^π^_ term in phenanthrenePyr_AntPyr_^AntPyr^ is more favorable for the linear than for the kinked isomer. Therefore, π-interactions are stronger in the most stable linear topology. However, relaxation of phenanthrenePyr_AntPyr_^AntPyr^ to phenanthrenePyr generates a slightly more stable kinked structure because of the large reduction in Pauli repulsions.

**Table 5:** Energy decomposition analysis (EDA) data for pyrilium derivatives of anthracene and phenanthrene (in kcal mol^−1^), and the corresponding turn-upside-down derivative.

	**AnthracenePyr_AntPyr_^AntPyr^**	**PhenanthrenePyr_PhePyr_^PhePyr^**		**PhenanthrenePyr_AntPyr_^PhePyr^**		**PhenanthreneO2_AntPyr_^AntPyr^**
	**AntPyr**+**AntPyr'**	**PhePyr**+**PhePyr**	*ΔΔ**E***	**AntPyr**+**AntPyr**	*ΔΔ**E***	**AntPyr**+**AntPyr**	*ΔΔ**E***
ΔE_Pauli_	510.3	487.1	−23.2	496.4	−13.9	516.9	6.6
ΔV_elstat_	−228.1	−214.5	13.6	−217.3	10.8	−227.1	1.0
ΔE_oi^σ^_	−405.9	−403.9	2.0	−407.1	−1.2	−415.0	−9.1
ΔE_oi^π^_	−103.7	−98.2	5.5	−98.2	5.5	−100.1	3.5
ΔE_oi_	−509.6	−502.1	7.5	−505.3	4.3	−515.1	−5.6
ΔE_disp_	−8.1	−9.1	−1.0	−9.1	−1.1	−9.3	−1.2
ΔE_int_	−235.5	−238.7	−3.2	−235.3	0.2	−234.7	0.8
ΔE_strain_	17.0	19.2	2.2	17.1	0.2	17.1	0.2
ΔE	−218.5	−219.4	−0.9	−218.2	0.3	−217.5	0.9

### Qtaim

**Figure 5:**
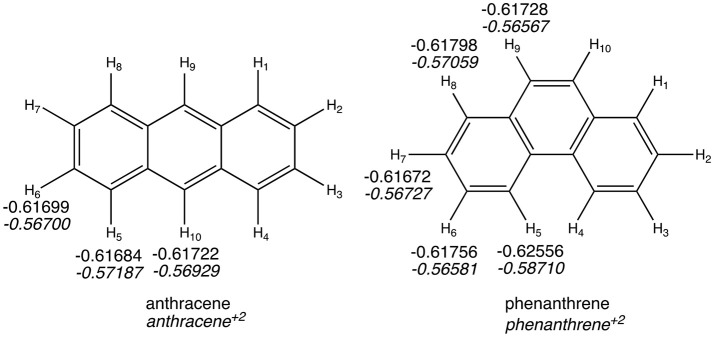
Atoms-in-Molecules (AIM) hydrogen atomic energies (in a.u.). In italics, values for dicationic species.

The QTAIM atomic energies of the H atoms involved in the H···H interaction in the bay region of phenanthrene were calculated for the neutral and dicationic systems (see Figure 5). In case of the neutral isomers, the H atoms in the bay region of phenanthrene are stabilized by −5.2 kcal mol^−1^ compared to the non-interacting hydrogen atoms in linear anthracene. This stabilization was used to justify the larger stability of the kinked isomers (Matta et al., 2003), as stated above. However, in case of the dicationic systems, the same H atoms in phenanthrene dication are even more stabilized, by −11.2 kcal mol^−1^, compared the non-interacting hydrogen atoms in dicationic anthracene. This should translate into a more stable charged phenanthrene than anthracene, which is not the case, thus providing an additional evidence that QTAIM atomic energies of few selected atoms cannot be used to justify isomerization energies. In addition, the same trend is found for the tetramethylated-pyrano-chromene derivatives, for which the bay H atoms in the kinked isomer are −3.0 kcal mol^−1^ more stable than in the linear one. Again, this result does not concur with the greater stability of the linear isomer.

## Conclusions

The main conclusion of this work is that the relative stability of kinked vs. straight topologies in polycyclic aromatic hydrocarbons (PAHs) depends on the aromaticity of the rings of the isomers. So, the answer to the question whether kinked is more stable than straight depends on the aromaticity of the two isomers. Phenanthrene is more stable than anthracene due to the larger stability of the π-system of the former, which is more aromatic. When two electrons are removed, i.e., dicationic systems are analyzed, the reverse trend is obtained, so the linear isomer is more stable than the kinked one. The larger stability of the former also correlates with its larger aromaticity, despite the difference in aromaticity between the isomers is now smaller. Finally, tetramethylated-pyrano-chromene isomers almost present the same aromatic character, whereas pyrilium derived systems show divergent aromaticities between central and terminal rings, i.e., central is more aromatic for the linear and terminal for the kinked. In these cases, when no isomer is clearly more aromatic, the relative stability is determined by the balance between the lower steric repulsion of the kinked vs. the more favorable electrostatic and orbital interactions of the linear.

Just to conclude, our study together with other works (Poater et al., 2006a,b, 2007a,b, 2017) clearly contradicts previous studies based on QTAIM analysis stating that the larger stability of phenanthrene is due to the stability of the H···H bridging interaction (Matta et al., 2003). This interaction is more stable for the kinked isomer in the three sets of isomers analyzed; however only in one case, the kinked system is more stable.

## Author contributions

JP and MS conceived and designed research. JP, MD, and MS analyzed data, interpreted results and wrote the paper. JP performed the calculations.

### Conflict of interest statement

The authors declare that the research was conducted in the absence of any commercial or financial relationships that could be construed as a potential conflict of interest.
