# Supplementary material for: Aromaticity Determines the Relative Stability of Kinked vs. Straight Topologies in Polycyclic Aromatic Hydrocarbons
Source: Front Chem. 2018 Nov 20;6:561. doi: 10.3389/fchem.2018.00561 (PMC6255896; doi:10.3389/fchem.2018.00561)

***SUPPLEMENTARY MATERIAL***

**Aromaticity determines the relative stability of kinked vs. straight topologies in polycyclic aromatic hydrocarbons**

***Jordi Poater^1,2*^, Miquel Duran^3^ and Miquel Solà^3*^***

*^1^ ICREA, Pg. Lluís Companys 23, 08010 Barcelona, Spain,^2^ Departament de Química Inorgànica i Orgànica & IQTCUB, Universitat de Barcelona, Martí i Franquès 1-11, 08028 Barcelona, Catalonia, Spain, ^3^ Institut de Química Computacional i Catàlisi (IQCC) and Departament de Química, Universitat de Girona, C/ Maria Aurèlia Capmany 69, 17003 Girona, Catalonia, Spain*

**CONTENTS**

1. Cartesian coordinates (in Å) and electronic energies (in Hartrees) of all linear and kinked acenes under analysis. **Table S1**
2. NICS (in ppm) for anthracene and phenanthrene and derived species. **Table S2**
3. Anisotropy of the Current Induced Density (ACID) plots (isosurface = 0.040) at the B3LYP/6-311++G(3df,3pd) level of theory. **Figure S1**
4. Singly-occupied molecular orbitals (SOMOs) for neutral and dicationic anthracene and phenanthrene fragments. **Figure S2**
5. π molecular orbitals for neutral and dicationic anthracene and phenanthrene systems. **Figure S3**
6. VDD charges (in me^-^) of the atoms forming the bonds for each fragment. **Figure S4**
7. Singly-occupied molecular orbitals (SOMOs) for tetrametylated-pyrano-chromenes fragments. **Figure S5**
8. π molecular orbitals for tetrametylated-pyrano-chromene systems. **Figure S6**

**Table S1 | Cartesian coordinates (in Å) and electronic energies (in Hartrees) of all linear and kinked acenes under analysis.**

Phenanthrene (E = -539.675372, 0 imag. freq.)

C 0.000000 0.678575 2.091948

C 0.000000 0.728270 -0.379122

C 0.000000 1.421440 0.865381

C 0.000000 -0.678575 2.091948

C 0.000000 -3.556670 -0.296963

C 0.000000 -2.878126 -1.528100

C 0.000000 -1.497762 -1.564434

C 0.000000 -0.728270 -0.379122

C 0.000000 -1.421440 0.865381

C 0.000000 -2.834599 0.877152

C 0.000000 2.834599 0.877152

H 0.000000 -4.640370 -0.273157

H 0.000000 -3.440138 -2.455177

H 0.000000 -1.005367 -2.528126

C 0.000000 1.497762 -1.564434

H 0.000000 -3.346347 1.833984

C 0.000000 2.878126 -1.528100

C 0.000000 3.556670 -0.296963

H 0.000000 -1.228340 3.027307

H 0.000000 3.346347 1.833984

H 0.000000 1.005367 -2.528126

H 0.000000 3.440138 -2.455177

H 0.000000 4.640370 -0.273157

H 0.000000 1.228340 3.027307

Anthracene (E = -539.667226, 0 imag. freq.)

C 0.000000 0.000000 1.401972

C 0.000000 0.000000 -1.401972

C 0.000000 1.221968 -0.721575

C 0.000000 1.221968 0.721575

C 0.000000 -3.655719 0.712456

C 0.000000 -3.655719 -0.712456

C 0.000000 -2.476916 -1.405300

C 0.000000 -1.221968 -0.721575

C 0.000000 -1.221968 0.721575

C 0.000000 -2.476916 1.405300

C 0.000000 2.476916 -1.405300

C 0.000000 2.476916 1.405300

H 0.000000 -4.600052 1.244889

H 0.000000 -4.600052 -1.244889

H 0.000000 -2.476390 -2.490318

C 0.000000 3.655719 -0.712456

C 0.000000 3.655719 0.712456

H 0.000000 4.600052 -1.244889

H 0.000000 4.600052 1.244889

H 0.000000 0.000000 -2.487839

H 0.000000 2.476390 -2.490318

H 0.000000 2.476390 2.490318

H 0.000000 -2.476390 2.490318

H 0.000000 0.000000 2.487839

Phenanthrene^+2^ (E = -538.949063, 0 imag. freq.)

C 0.000000 0.722266 2.032225

C 0.000000 0.737658 -0.416103

C 0.000000 1.435152 0.854785

C 0.000000 -0.722266 2.032225

C 0.000000 -3.618383 -0.266100

C 0.000000 -2.932311 -1.485299

C 0.000000 -1.507553 -1.559144

C 0.000000 -0.737658 -0.416103

C 0.000000 -1.435152 0.854785

C 0.000000 -2.882965 0.898053

C 0.000000 2.882965 0.898053

H 0.000000 -4.701137 -0.244254

H 0.000000 -3.493607 -2.414574

H 0.000000 -1.060942 -2.545160

C 0.000000 1.507553 -1.559144

H 0.000000 -3.373096 1.866131

C 0.000000 2.932311 -1.485299

C 0.000000 3.618383 -0.266100

H 0.000000 -1.238515 2.987348

H 0.000000 3.373096 1.866131

H 0.000000 1.060942 -2.545160

H 0.000000 3.493607 -2.414574

H 0.000000 4.701137 -0.244254

H 0.000000 1.238515 2.987348

Anthracene^+2^ (E = -538.975211, 0 imag. freq.)

C 0.000000 0.000000 1.408954

C 0.000000 0.000000 -1.408954

C 0.000000 1.233310 -0.719307

C 0.000000 1.233310 0.719307

C 0.000000 -3.677571 0.693215

C 0.000000 -3.677571 -0.693215

C 0.000000 -2.457087 -1.406509

C 0.000000 -1.233310 -0.719307

C 0.000000 -1.233310 0.719307

C 0.000000 -2.457087 1.406509

C 0.000000 2.457087 -1.406509

C 0.000000 2.457087 1.406509

H 0.000000 -4.614416 1.238690

H 0.000000 -4.614416 -1.238690

H 0.000000 -2.470895 -2.491592

C 0.000000 3.677571 -0.693215

C 0.000000 3.677571 0.693215

H 0.000000 4.614416 -1.238690

H 0.000000 4.614416 1.238690

H 0.000000 0.000000 -2.495690

H 0.000000 2.470895 -2.491592

H 0.000000 2.470895 2.491592

H 0.000000 -2.470895 2.491592

H 0.000000 0.000000 2.495690

PhenanthreneO2 (E = -613.860939, 0 imag. freq.)

C 0.000000 0.696539 2.047194

C 0.000000 0.706544 -0.380284

C 0.000000 1.393096 0.845827

C 0.000000 -0.696539 2.047194

C 0.000000 -3.593355 -0.235685

C 0.000000 -2.850476 -1.534447

C 0.000000 -1.516433 -1.597030

C 0.000000 -0.706544 -0.380284

C 0.000000 -1.393096 0.845827

O 0.000000 -2.761640 0.935197

O 0.000000 2.761640 0.935197

H 0.000000 3.456534 -2.434372

H 0.000000 -3.456534 -2.434372

H 0.000000 -1.029600 -2.563577

C 0.000000 1.516433 -1.597030

H 0.000000 1.029600 -2.563577

C 0.000000 2.850476 -1.534447

C 0.000000 3.593355 -0.235685

H 0.000000 -1.252972 2.976227

H 0.000000 1.252972 2.976227

H -0.881635 4.244376 -0.166654

H 0.881635 4.244376 -0.166654

H -0.881635 -4.244376 -0.166654

H 0.881635 -4.244376 -0.166654

AnthraceneO2 (E = -613.867630, 0 imag. freq.)

C 0.000000 0.000000 -1.336577

C 0.000000 0.000000 1.446060

C 0.000000 1.222033 0.772639

C 0.000000 1.201904 -0.634971

C 0.000000 -3.647022 -0.767960

C 0.000000 -3.649236 0.731123

C 0.000000 -2.517224 1.439720

C 0.000000 -1.222033 0.772639

C 0.000000 -1.201904 -0.634971

O 0.000000 -2.340460 -1.381079

C 0.000000 2.517224 1.439720

O 0.000000 2.340460 -1.381079

H 0.000000 0.000000 -2.418567

H 0.000000 -4.622816 1.209303

H 0.000000 -2.540849 2.524530

C 0.000000 3.649236 0.731123

C 0.000000 3.647022 -0.767960

H 0.000000 4.622816 1.209303

H 0.000000 2.540849 2.524530

H 0.000000 0.000000 2.532369

H -0.881652 -4.172250 -1.156928

H 0.881652 -4.172250 -1.156928

H -0.881652 4.172250 -1.156928

H 0.881652 4.172250 -1.156928

**Table S2 | NICS (in ppm) for anthracene and phenanthrene and derived species.**

|  | ***central ring*** | | | | ***terminal ring*** | | | |
| --- | --- | --- | --- | --- | --- | --- | --- | --- |
|  | **NICS(0)** | **NICS(0)_zz_** | **NICS(1)** | **NICS(1)_zz_** | **NICS(0)** | **NICS(0)_zz_** | **NICS(1)** | **NICS(1)_zz_** |
| **anthracene** | -11.4 | -19.1 | -12.8 | -34.8 | -7.3 | -9.8 | -9.5 | -25.9 |
| **phenanthrene** | -5.6 | -2.2 | -8.3 | -20.6 | -8.5 | -13.0 | -10.7 | -28.8 |
|  |  |  |  |  |  |  |  |  |
| **anthracene^+2^** | 16.5 | 61.0 | 9.7 | 34.4 | 11.1 | 42.0 | 4.7 | 18.3 |
| **phenanthrene^+2^** | 23.8 | 82.1 | 16.1 | 53.6 | 26.8 | 88.9 | 17.9 | 58.6 |
|  |  |  |  |  |  |  |  |  |
| **anthraceneO2** | -6.7 | 1.3 | -6.7 | -14.7 | 4.2 | 33.4 | 2.3 | 9.7 |
| **phenanthreneO2** | 1.2 | 20.8 | -1.9 | -1.1 | 2.8 | 17.6 | -2.5 | -1.4 |

**Figure S1 | Anisotropy of the Current Induced Density (ACID) plots (isosurface = 0.040) at the B3LYP/6-311++G(3df,3pd) level of theory.**


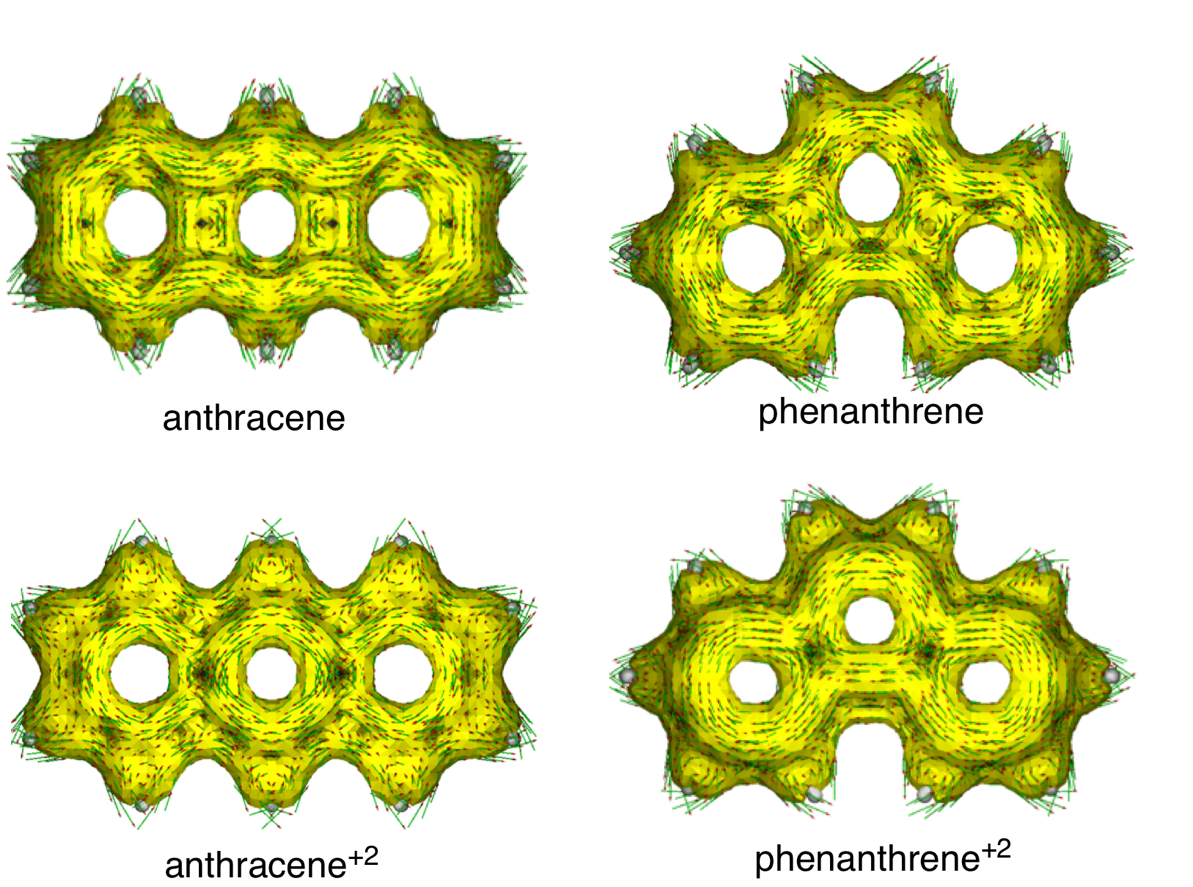


**Figure S2 | Singly-occupied molecular orbitals (SOMOs) for neutral and dicationic anthracene and phenanthrene fragments.**


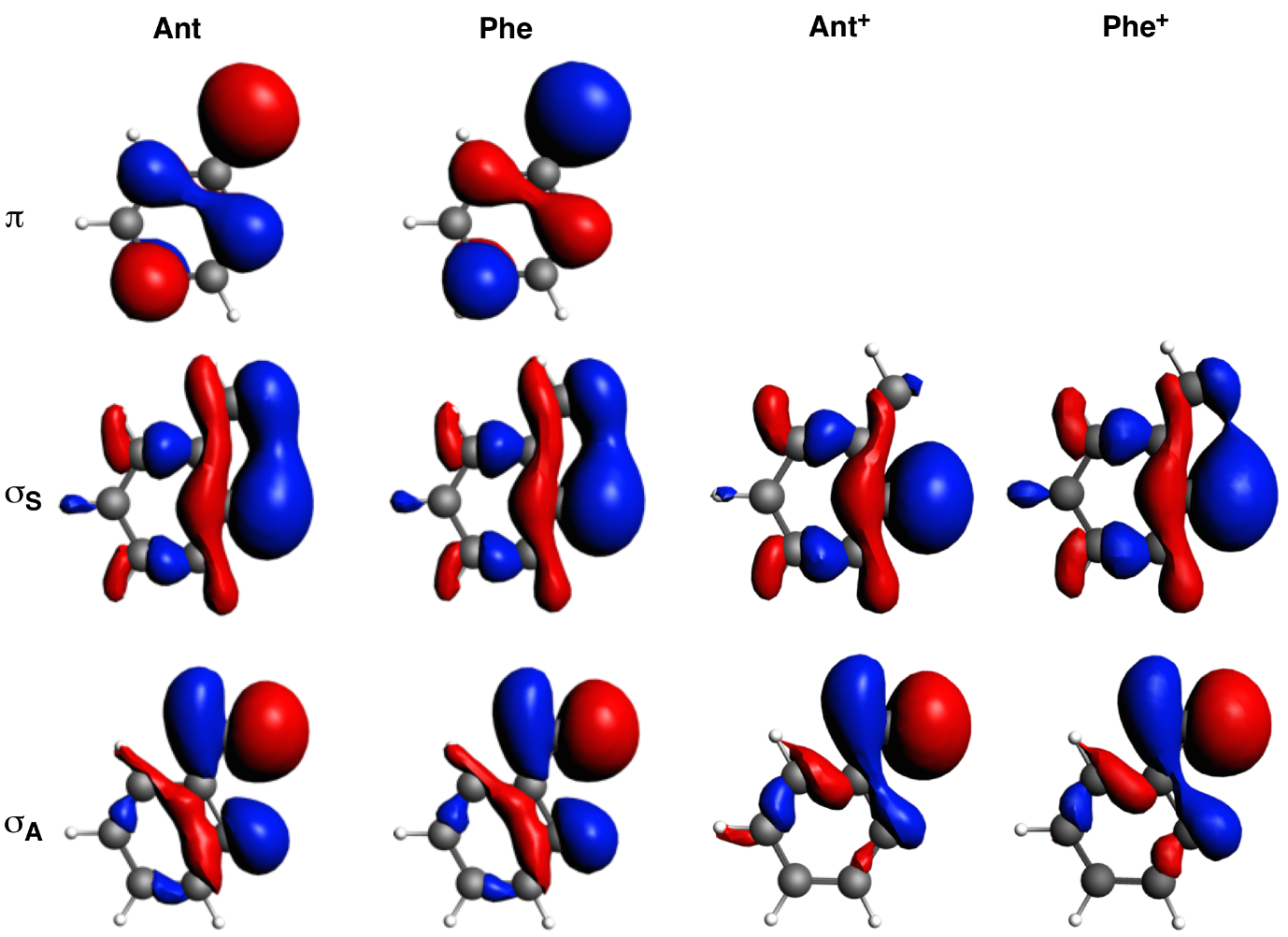


**Figure S3 | π molecular orbitals for neutral and dicationic anthracene and phenanthrene systems.**


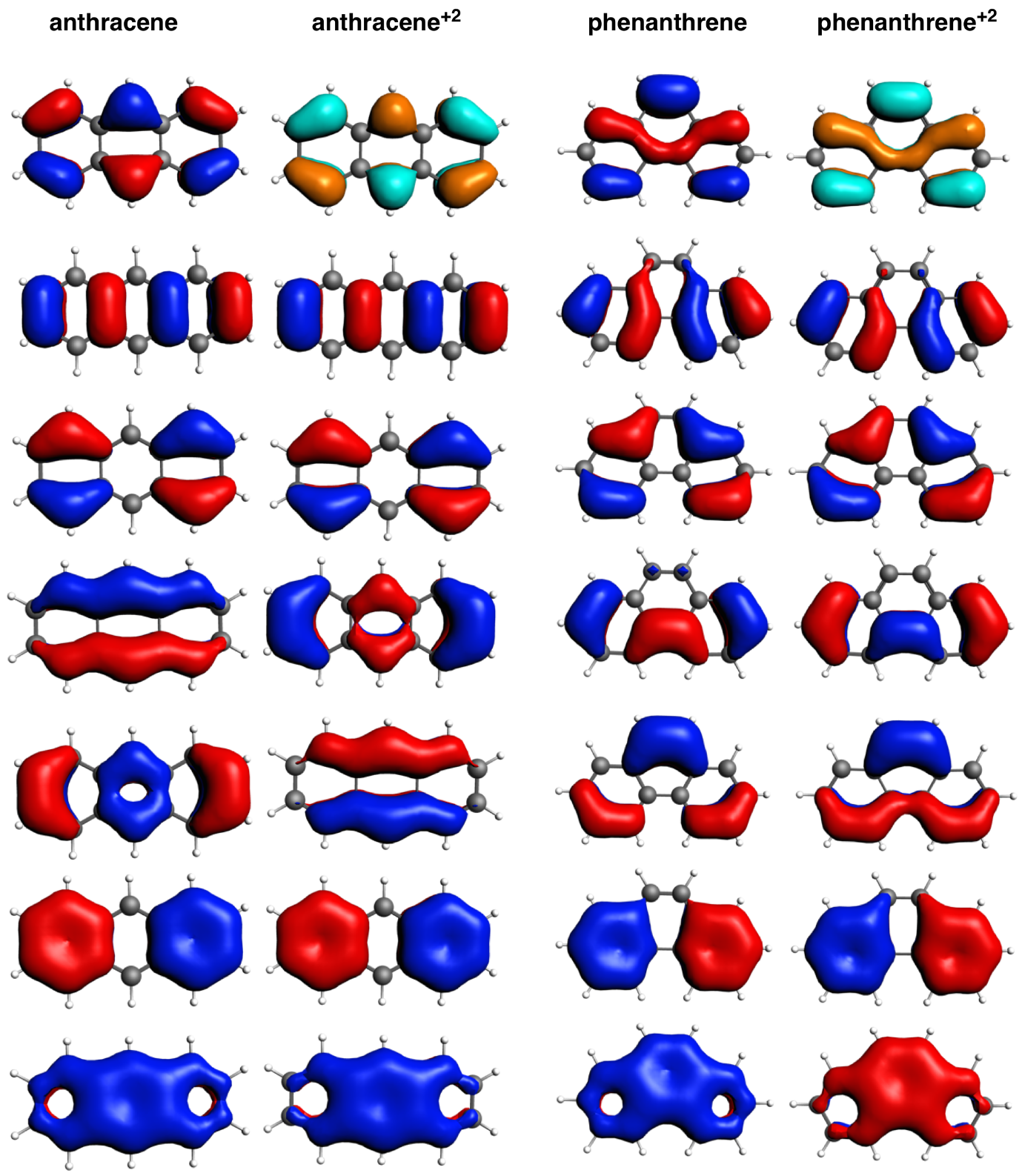


**Figure S4 | VDD charges (in me^-^) of the atoms forming the bonds for each fragment.**

**Figure S5 | Singly-occupied molecular orbitals (SOMOs) for tetrametylated-pyrano-chromenes fragments.**


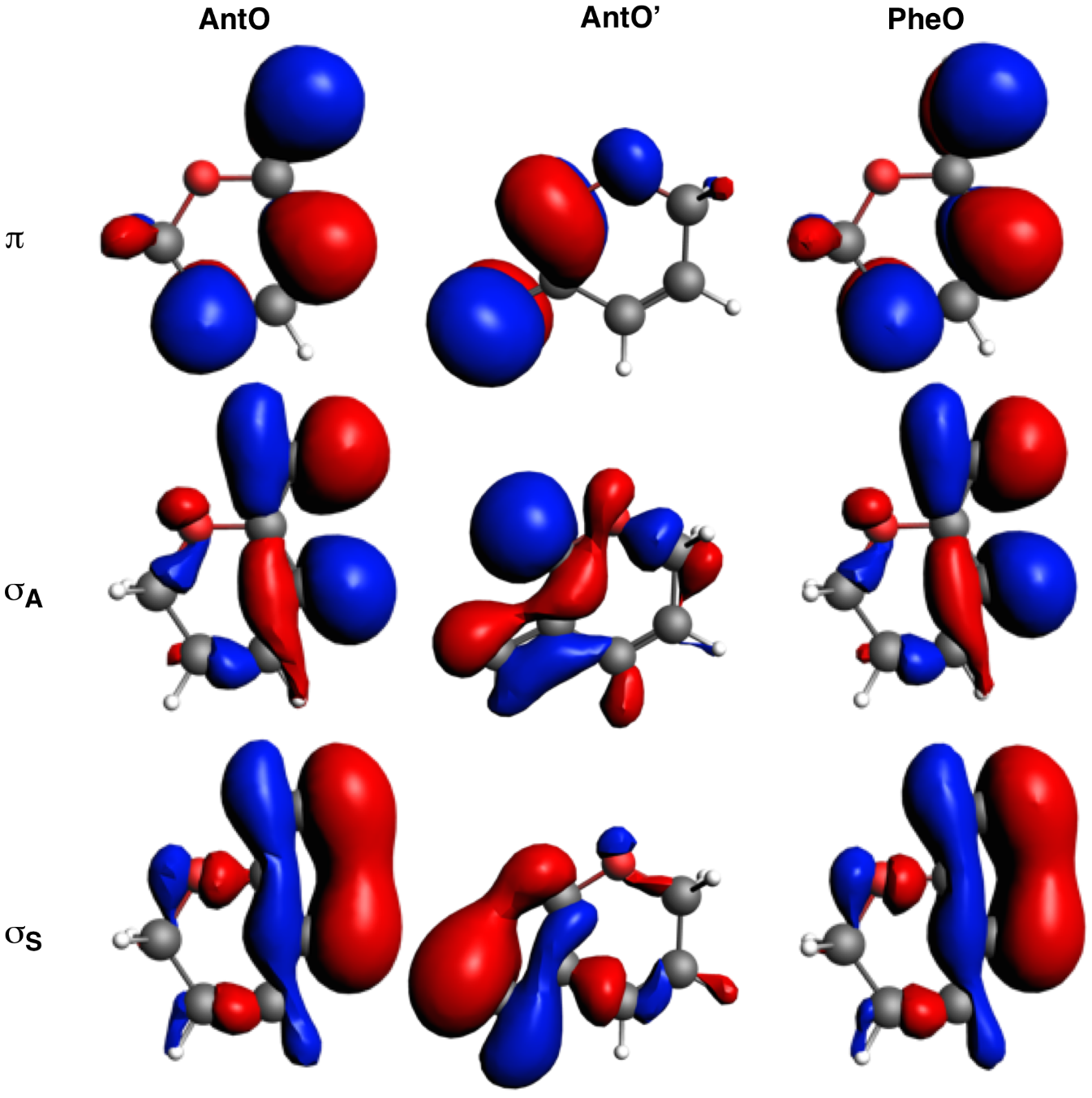


**Figure S6 | π molecular orbitals for tetrametylated-pyrano-chromene systems.**


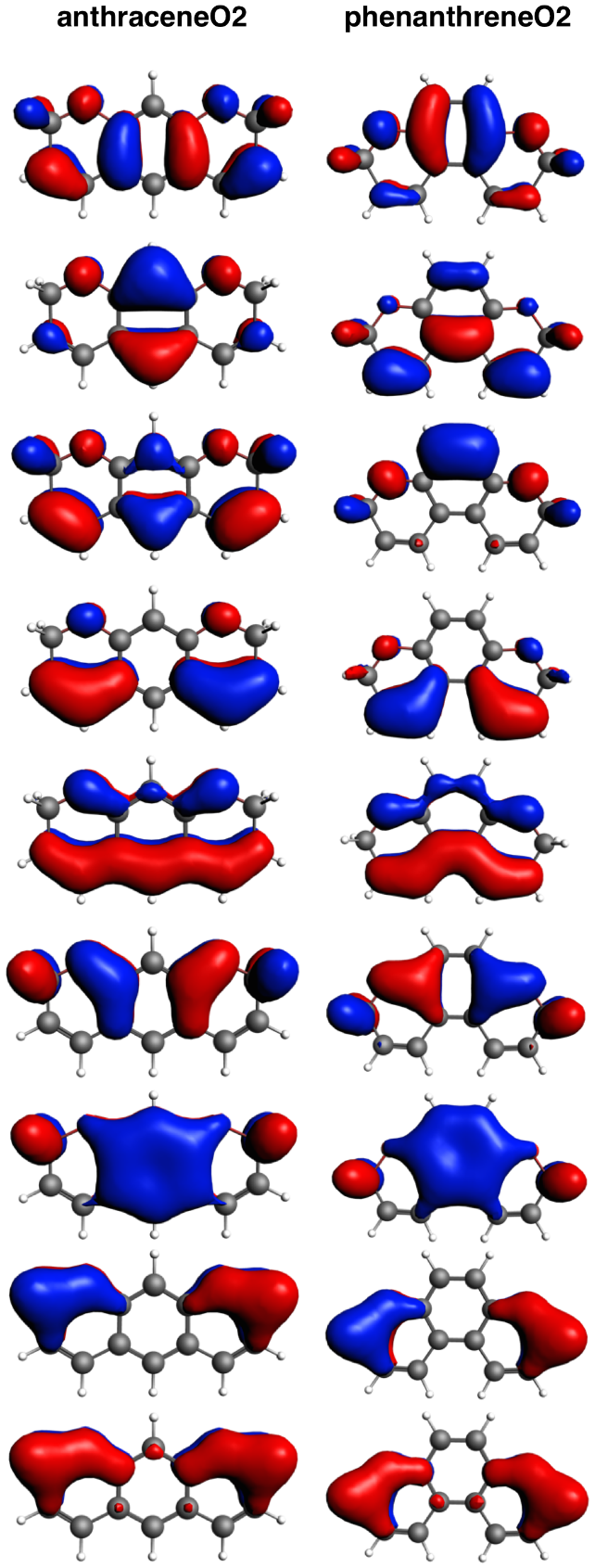

Supplement: Supplementary file 1 [file Table_1.DOCX]
